# In Silico Identification of miRNA–lncRNA Interactions in Male Reproductive Disorder Associated with COVID-19 Infection

**DOI:** 10.3390/cells10061480

**Published:** 2021-06-12

**Authors:** Soudabeh Sabetian, Isabella Castiglioni, Bahia Namavar Jahromi, Pegah Mousavi, Claudia Cava

**Affiliations:** 1 Infertility Research Center, Shiraz University of Medical Sciences, Shiraz, Iran; soudabehsabet@gmail.com (S.S.); namavarb@sums.ac.ir (B.N.J.); 2 Department of Physics “Giuseppe Occhialini”, University of Milan-Bicocca Piazza dell’Ateneo Nuovo, 20126 Milan, Italy; 3 Department of Obstetrics and Gynecology, School of Medicine, Shiraz University of Medical Sciences, Shiraz, Iran; 4 Department of Medical Genetics, Faculty of Medicine, Hormozgan University of Medical Sciences, Bandar Abbas, Iran; pegahmousavi2017@gmail.com; 5 Institute of Molecular Bioimaging and Physiology, National Research Council (IBFM-CNR), Via F.Cervi 93, Segrate, 20090 Milan, Italy

**Keywords:** COVID-19, male infertility, lncRNA, miRNA, interactions

## Abstract

Coronavirus disease 2019 (COVID-19), a global pandemic, is caused by severe acute respiratory syndrome coronavirus 2 (SARS-CoV-2). Angiotensin-converting enzyme 2 (ACE2) is the receptor for SARS-CoV-2 and transmembrane serine protease 2 (TMPRSS2) facilitates ACE2-mediated virus entry. Moreover, the expression of *ACE2* in the testes of infertile men is higher than normal, which indicates that infertile men may be susceptible to be infected and SARS-CoV-2 may cause reproductive disorder through the pathway induced by *ACE2* and *TMPRSS2*. Little is known about the pathway regulation of *ACE2* and *TMPRSS2* expression in male reproductive disorder. Since the regulation of gene expression is mediated by microRNAs (miRNAs) and long non-coding RNAs (lncRNAs) at the post-transcriptional level, the aim of this study was to analyze the dysregulated miRNA–lncRNA interactions of *ACE2* and *TMPRSS2* in male reproductive disorder. Using bioinformatics analysis, we speculate that the predicted miRNAs including *miR-125a-5p*, *miR-125b-5p*, *miR-574-5p*, and *miR-936* as regulators of *ACE2* and *miR-204-5p* as a modulator of *TMPRSS2* are associated with male infertility. The lncRNAs with a tissue-specific expression for testis including GRM7-AS3, ARHGAP26-AS1, BSN-AS1, KRBOX1-AS1, CACNA1C-IT3, AC012361.1, FGF14-IT1, AC012494.1, and GS1-24F4.2 were predicted. The identified miRNAs and lncRNAs are proposed as potential biomarkers to study the possible association between COVID-19 and male infertility. This study encourages further studies of miRNA–lncRNA interactions to explain the molecular mechanisms of male infertility in COVID-19 patients.

## 1. Introduction

In December 2019, a novel coronavirus responsible for coronavirus disease 2019 (COVID-19) was described in Wuhan, China [1,2]. To date, nearly 146,000,000 global confirmed cases of COVID-19, with nearly 3 million deaths, have been observed [3].

SARS-CoV-2, a single-stranded RNA virus belonging to the coronavirus subfamily, is one of the seven different coronaviruses that can infect humans and is the pathogen responsible for COVID-19 [4]. Four coronaviruses, 229E, NL63, OC43, and HKU1, can lead to mild viral symptoms, while the other three, SARS-CoV-1, MERS-CoV, and SARS-CoV-2, can cause more severe respiratory symptoms [4].

The angiotensin-converting enzyme 2 (*ACE2*) receptor and transmembrane serine protease 2 (*TMPRSS2*), localized on the host cell membrane, are indispensable for viral proliferation in the infected host [5].

*ACE2* and *TMPRSS2* are highly expressed in normal human tissues, such as the lung, heart, colon, and testis [6,7]. Overall, the epidemiological findings reported that males are more vulnerable to the infection than females [8]. However, it seems there is not a clear association between the localization of receptors and virus infectivity.

*ACE2* and *TMPRSS2* are androgen-regulated and expressed in both germ cells and somatic cells [9,10,11].

Previous studies measured hormonal levels in COVID-19-infected male patients and they found a lower expression of testosterone levels and higher luteinizing hormone levels, key mediators in male reproductive health [12,13].

Li et al. indicated that SARS-CoV-2 can be present in the semen of patients with COVID-19 [14]. Ma and colleagues confirmed the nucleic acid sequence of SARS-CoV-2 using RT-qPCR and the presence of the virus by immunohistochemistry in the testes of COVID-19 patients. Furthermore, they demonstrated a higher expression of *ACE2* and *TMPRSS2* in the testes of infertile men than normal [15]. The SARS-CoV-2 spike protein binds to the ACE2 receptor of the target cells and TMPRSS2 primes cellular protease to cleave the S protein into S1 and S2 subunits. The two subunits have two different roles: S1 is the domain where there is the binding with the ACE2 receptor and S2 is responsible for the fusion with the target cell membrane [5]. Collectively, the previous findings indicate that men with reproductive disorders may be easily infected by SARS-CoV-2 and this virus may cause male reproductive disorders through the pathway activated by *ACE2* and *TMPRSS2* [16,17].

Despite these recent studies, there are currently no biomarkers able to establish the effects of COVID-19 and elucidate the molecular mechanisms associated with male infertility. Therefore, non-invasive approaches that can diagnose the effects of COVID-19 on male infertility are appealing aspects [18].

The regulation of gene expression is mediated by micro RNAs (miRNAs) and long non-coding RNAs (lncRNAs) at the post-transcriptional level in multiple molecular mechanisms. miRNAs, small non-coding RNAs, are important regulators of gene expression through binding to the 3′ untranslated region of their complementary mRNA sequences and lead to their degradation or inhibition of translation [19,20,21]. Previous studies reported the significant role of miRNAs in spermatogenesis and testicular development [22,23]. As miRNAs are abundant in plasma, serum, and seminal plasma, it makes them appealing potential non-invasive biomarkers [24]. Indeed, miRNAs can modify the host’s transcriptome and modulate viral infection through the regulation of biological pathways with pro- or antiviral effects [25].

lncRNAs, sequences of RNA longer than 200 nucleotides that are not translated into proteins [26], can sponge miRNAs to moderate their regulatory effect on mRNAs; the association between lncRNAs and miRNAs is essential for gene regulation [27]. Various lncRNAs are involved in modulating mammalian spermatogenesis [28]. Changes in miRNA and lncRNA expression profiles in patients with non-obstructive azoospermia supported their role in male infertility [20]. In line with this scenario, miRNAs and lncRNAs could also have potential therapeutic applications. Nevertheless, the molecular mechanism of miRNAs and lncRNAs on the course of male infertility associated with COVID-19 remains poorly understood.

In this study, the expression profiles of miRNAs and lncRNAs which regulate *ACE2* “the hottest targets of SARS-CoV-2” and *TMPRSS2* “S protein priming” were analyzed comprehensively in infertile men by bioinformatics approaches. We proposed potential biomarkers which might help to understand the effects of COVID-19 on male infertility.

## 2. Materials and Methods

### 2.1. Predicting the Interactions miRNA–ACE2 and miRNA–TMPRSS2

The miRNAs that target *ACE2* and *TMPRSS2* were predicted by mirDIP database Version 4.1.0.3 [29]. This integrated database covers 30 datasets including TargetScan, RNAhybrid, mirTar, mirbase, DIANA, etc. [29]. The score class was limited to high and very high interactions. 

### 2.2. Screening the miRNAs Associated with Male Infertility

In order to filter the retrieved miRNAs associated with male infertility, differentially expressed miRNAs (DE-miRNAs) in human testes from infertile men were obtained from the study of Abu et al. [30]. |Log FC| > 3 and *p* value < 0.05 were considered statistically significant to identify differentially expressed miRNAs. 

### 2.3. Predicting the Interactions lncRNAs–miRNAs

The identified DE-miRNAs were submitted to miRWalk2 [31] to predict the association between miRNA and lncRNA.

### 2.4. Filtering the lncRNAs Associated with Male Infertility

**Figure 1:**
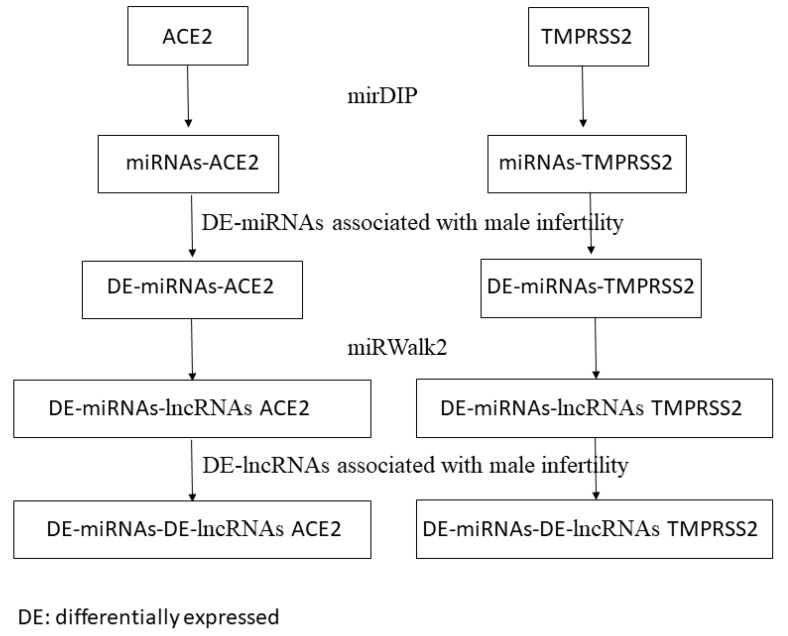
Workflow of the proposed computational approach. DE: differentially expressed in infertile men; DE: differentially expressed.

The predicted lncRNAs in the last step were screened according to lncRNAs associated with male infertility that have been identified by Joshi and Lu [32,33]. |Log FC| > 3 and *p* value < 0.05 were considered statistically significant to identify differentially expressed lncRNAs. The computational procedure of our study is represented in Figure 1. miRNAs able to regulate *ACE2* and *TMPRSS2* and associated with male infertility were identified through mirDIP and the study of Abu et al. [30].

In addition, we identified the interactions between these miRNAs and differentially expressed lncRNAs in infertile men identified by Joshi and Lu [32,33].

### 2.5. Gene Ontology Analysis

Gene Ontology (GO) and panther version 16.0 were used to perform functional classification of identified lncRNAs in categories such as molecular function, biological process and cellular component [34].

### 2.6. Testis-Specific lncRNAs

The Gini index was used to explore testis-specific lncRNAs. The Gini index was calculated to evaluate the specificity of expression of lncRNAs compared to different healthy tissues. We used the public database GTEX that contains the expression levels in 30 human normal tissues: adipose tissue, adrenal gland, bladder, blood, blood vessel, brain, breast, cervix uteri, colon, esophagus, fallopian tube, heart, kidney, liver, lung, muscle, nerve, ovary, pancreas, pituitary, prostate, salivary gland, skin, small intestine, spleen, stomach, testis, thyroid, uterus, and vagina [35]. We analyzed the Gini index for each normal tissue and quantified the specificity of lncRNAs for testis. It can assume a value from 0 to 1. We defined a lncRNA specific for testis if the Gini index is <= 0.15. We considered 10167 lncRNAs in GTEX data as reported from the lncRNome database [36].

## 3. Results

### 3.1. miRNAs Associated with Male Infertility Regulate ACE2 and TMPRSS2

From our bioinformatics analysis we predicted that 80 miRNAs and 92 miRNAs can regulate *ACE2* and *TMPRSS2*, respectively. Ten miRNAs (*miR-1208*, *miR-141-3p*, *miR-182-5p*, *miR-300*, *miR-331-3p*, *miR-362-5p*, *miR-381-3p*, *miR-4308*, *miR-582-5p* and *miR-587*) were found in common between *ACE2* and *TMPRSS2*.

Furthermore, we explored the miRNAs that regulate *ACE2* and *TMPRSS2* and are associated with male infertility according to the study of Abu et al. [30]. Four miRNAs (*miR-125a-5p*, *miR-125b-5p*, *miR-574-5p*, and *miR-936*) were associated with male infertility and were also predicted to modulate *ACE2* expression. *miR-204-5p*, with an effective role in male infertility, was presented as a possible regulator of *TMPRSS2*. Supplementary File 1 shows the list of all predicted miRNAs as regulators of *ACE2* and *TMPRSS2*, the miRNAs associated with male infertility, and the extracted miRNAs from the predicted ones which were also associated with male infertility.

### 3.2. lncRNAs–miRNAs Associated with Male Infertility

A total of 5612 lncRNAs were predicted to interact with *miR-125a-5p* and *miR-125b-5p*, and 3416 lncRNAs were predicted to interact with *miR-936*. *miR-204-5p* was proposed to have interactions with 6569 lncRNAs. 

We extracted 349 unique lncRNAs which were associated with male infertility. A total of 155 lncRNAs have interactions with *miR-125a-5p* and *miR-125b-5p*, 122 lncRNAs with *miR-936*, and 187 lncRNAs with *miR-204-5p*. Supplementary File 2 shows the list of 349 lncRNAs.

### 3.3. Functional Annotations

**Figure 2:**
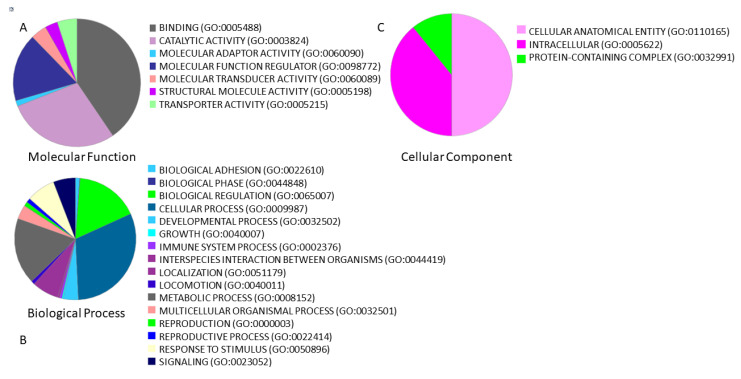
Functional classification using PANTHER of 349 lncRNAs associated with male infertility and interacting with *miR-125a-5p*, *miR-125b-5p*, *miR-936* and *miR-204-5p*. Overall, we found 349 unique lncRNAs. The percentage of lncRNAs involved in (**A**) molecular function, (**B**) biological processes, and (**C**) cellular component is represented by a pie chart.

The biological role of 349 lncRNAs was evaluated through a functional analysis. From the 349 unique lncRNAs, 323 lncRNAs were annotated by PANTHER and functional analysis of the lncRNAs showed that the top molecular functions (Figure 2a) were “bindings” (40.5% of lncRNAs) and “catalytic activity” (28.6% of lncRNAs); the top biological processes (Figure 2b) were “cellular process” (31% of lncRNAs) and “biological regulation” (16.9% of lncRNAs); and the top cellular components (Figure 2c) were “cellular anatomical entity” (50% of lncRNAs) and “intracellular” (39.2% of lncRNAs).

Biological process analysis demonstrated that the identified lncRNAs were involved in the immune system and reproductive process. 

### 3.4. Possible Roles of lncRNAs in Male Reproductive Disorder Associated with COVID-19

In order to investigate lncRNAs which could play a regulatory role in COVID-19, and especially its male reproductive system consequences, we performed an analysis to select testis-specific lncRNAs. We found that the expression levels of 9 of 349 lncRNAs (GRM7-AS3, ARHGAP26-AS1, BSN-AS1, KRBOX1-AS1, CACNA1C-IT3, AC012361.1, FGF14-IT1, AC012494.1, and GS1-24F4.2) were specific for testis tissue, demonstrating their possible crucial role in the male reproductive system.

GRM7-AS3, ARHGAP26-AS1, BSN-AS1, and KRBOX1-AS1 interact with *miR-936*, and CACNA1C-IT3, AC012361.1, FGF14-IT1, AC012494.1, and GS1-24F4.2 interact with *miR-204-5p*.

## 4. Discussion

In this study, we analyzed the potential association of the dysregulated miRNAs and lncRNAs in male infertility and their association with *ACE2* and *TMPRSS2*. First, we studied miRNAs that could regulate two crucial genes for viral proliferation in the infected host, *ACE2* and *TMPRSS2*. Furthermore, we selected those miRNAs that regulate *ACE2* and *TMPRSS2* and are associated with male infertility. We identified four miRNAs, *miR-125a-5p, miR-125b-5p, miR-574–5p*, and *miR-936*, that regulate *ACE2* and are differentially expressed in infertile men. *TMPRSS2* is regulated by *miR-204-5p*, a differentially expressed miRNA in infertile men.

A previous study demonstrated a potential role of *miR-125b-5p* in hepatitis B virus (HBV) and COVID-19. Indeed, *miR-125b-5p* and HBV DNA levels were positively associated, demonstrating its role in HBV infection [37]. Regarding COVID-19, *miR-125b-5p* could modify the risk of SARS-CoV-2 infection in lung cancer patients [38].

*miR-125a-5p* and *miR-125b-5p* were found to be over-expressed in Sertoli cells, and in the epididymis of fertile men [39,40]. Salas et al. analyzed miRNA profiles of patients with teratozoospermia and oligozoospermia and *miR-125a-3p* was found to be downregulated in both the conditions [41]. In addition, previous studies reported a potential interaction between *ACE2* and histone deacetylase, *HDAC2*, suggesting a regulatory network that involves *miR-125a–ACE2–HDAC2* [42]. Although the role of *HDAC2* in spermatogenesis is not completely understood, previous studies demonstrated that genes regulated by HDAC2 are involved in spermatogonial stem cells [43].

In our study, *miR-574–5p* was found to be down-expressed in Sertoli cells of infertile men. We can hypothesize that an increase in *ACE2* expression reported in infertile men with COVID-19 can be due to the downregulation of *miR-574–5p* [44]. In addition, *miR-574–5p* was also proposed as an agent with antiviral activity in HBV, downregulating the expression of HBV polymerase mRNA [45]. A possible mechanism that reinforces its possible role in COVID-19 treatment is reported by a recent study that showed that the upregulation of *miR-574–5p* inhibits TLR4/ NF-kB signaling and downregulates the production of proinflammatory cytokines in patients with acute respiratory distress syndrome [46]. In line with this scenario, *miR-574–5p* could reduce the cytokine storm, one of the most common causes of death in patients with COVID-19 [46]. Proinflammatory cytokines are important regulators of testis development and male fertility, suggesting a complex regulatory network of *miR-574–5p*–*ACE2*–TLR4/NF-kB–cytokines. 

*miR-936* is indicated as a regulator of *ACE2* in placentas [47]. In addition, a previous study showed that a fibroblast growth factor, *FGF2*, is a direct target of *miR-936* [48]. In a previous study on Zika virus, the inhibition of *FGF2* affected viral replication through the inhibition of the MAPK pathway, which is associated with normal FGF/FGFR activity [49]. *FGF2* seems to also play a crucial role in male reproductive tissues [50]. In addition, several studies demonstrated the positive correlation between *FGF2* and angiotensin, suggesting a small regulatory circuit that involves *miR-936–ACE2*–*FGF2* in COVID-19 and male infertility [51].

In our study, we found that *TMPRSS2* is regulated by *miR-204-5p. miR-204* was found to be over-expressed in prostate cancer cell lines compared to human prostate tissue. In prostate cancer, different genomic rearrangements can occur, such as the most common fusion of androgen receptor *TMPRSS2* with *ERG*. *miR-204* is a TMPRSS2/ERG oncofusion negative regulator and can act as a tumor suppressor or oncomiR, regulating the genes under androgen receptor control [52]. In addition, *miR−204b−5p* was found to be abundant in the spermatozoa of the epididymis, suggesting its crucial role in the male reproductive system [53].

Furthermore, we explored the interactions between differentially expressed lncRNAs in infertile men and the miRNAs reported above. From this analysis, we identified 349 lncRNAs as potential biomarkers explaining the potential effects of COVID-19 on the male reproductive system. The role of 349 lncRNAs was analyzed with a pathway analysis and we found that they are involved mainly in regulation and binding.

In order to define a selected number of lncRNAs, we evaluated the testis-specific lncRNAs in the 349 lncRNAs. We found that 9 out of 349 lncRNAs were testis specific: GRM7-AS3, ARHGAP26-AS1, BSN-AS1, and KRBOX1-AS1 interacting with *miR-936*, and CACNA1C-IT3, AC012361.1, FGF14-IT1, AC012494.1, and GS1-24F4.2 interacting with *miR-204-5p*. Little is known about the role of these lncRNAs. GRM7-AS3 and KRBOX1-AS1 are more known and studied in the literature.

GRM7-AS3 is complementary to a functional RNA, GRM7. Currently, there are no studies that reported an association between GRM7-AS3, COVID-19 and the male reproductive system. A previous study reported that GRM7 plays a role in neurologic diseases such as depression, epilepsy and bipolar disorder, regulating synaptic activity [54].

KRBOX1-AS1 has also been correlated with programmed cell death and cell proliferation in rectal cancer [55]. Although KRBOX1 was found to be over-expressed in testis by a previous study, its role has not been investigated [56].

As these lncRNAs were found to be differentially expressed in infertile men and their expression in healthy men was testis-specific, we proposed these lncRNAs as biomarkers that could explain the association between COVID-19 and male reproductive disorder. miRNAs interacting with these lncRNAs (*miR-936* and *miR-204-5p*) could also play an important role in this molecular mechanism.

Overall, this study predicted the interactions of DE-miRNAs and DE-lncRNAs from infertile men with *ACE2* and *TMPRSS2* using bioinformatics approaches. We assumed the proposed miRNAs and lncRNAs can be potential biomarkers to examine the effect of SARS-CoV-2 on testis and spermatogenesis damages. However, further experimental study is required to confirm the current findings through comparing the infertile men and normal cases after COVID-19 infection.

## 5. Conclusions

miRNAs and lncRNAs are involved in various mechanisms of COVID-19 infection and male infertility, but their roles are not fully understood. The present study proposed the miRNAs and lncRNAs as possible diagnostic tools regarding the pathogenic role of SARS-CoV-2 in male infertility.

The miRNAs, including *miR-125a-5p, miR-125b-5p, miR-574–5p*, *miR-936* and *miR-204-5p*, and the associated lncRNAs, including GRM7-AS3, ARHGAP26-AS1, BSN-AS1, KRBOX1-AS1, CACNA1C-IT3, AC012361.1, FGF14-IT1, AC012494.1, and GS1-24F4.2, could shed a light on possible diagnostic applications in male infertility after SARS-CoV-2 infection. Further studies are encouraged to validate the current findings by comparing normal and infertile men after infection with COVID-19.

## Data Availability

Data supporting reported results can be found in publicly archived datasets: https://gtexportal.org/home/.

## Supplementary Materials

The supplementary files are available online at https://www.mdpi.com/article/10.3390/cells10061480/s1. Supplementary File 1 shows the list of gene targets of *ACE2* and *TMPRSS2*. Supplementary File 2 shows the list of 349 lncRNAs associated with male infertility and interacting with miRNAs associated with COVID-19 and male infertility.
